# European Bone Mineral Density Loci Are Also Associated with BMD in East-Asian Populations

**DOI:** 10.1371/journal.pone.0013217

**Published:** 2010-10-07

**Authors:** Unnur Styrkarsdottir, Bjarni V. Halldorsson, Daniel F. Gudbjartsson, Nelson L. S. Tang, Jung-Min Koh, Su-mei Xiao, Timothy C. Y. Kwok, Ghi Su Kim, Juliana C. N. Chan, Stacey Cherny, Seung Hun Lee, Anthony Kwok, Suzanne Ho, Solveig Gretarsdottir, Jelena Pop Kostic, Stefan Th. Palsson, Gunnar Sigurdsson, Pak C. Sham, Beom-Jun Kim, Annie W. C. Kung, Shin-Yoon Kim, Jean Woo, Ping-C. Leung, Augustine Kong, Unnur Thorsteinsdottir, Kari Stefansson

**Affiliations:** 1 deCODE Genetics, Reykjavik, Iceland; 2 Reykjavik University, Reykjavik, Iceland; 3 Department of Chemical and Laboratory for Genetics of Disease Susceptibility, Faculty of Medicine, Li Ka Shing Institute of Health Sciences, The Chinese University of Hong Kong, Hong Kong, China; 4 Division of Endocrinology and Metabolism, University of Ulsan College of Medicine, Asan Medical Center, Seoul, Republic of Korea; 5 Skeletal Diseases Genome Research Center, Kyungpook National University Hospital, Daegu, Republic of Korea; 6 Department of Medicine and Research Centre of Heart, Brain, Hormone and Healthy Aging, The University of Hong Kong, Hong Kong, China; 7 Department of Medicine & Therapeutics, Faculty of Medicine, The Chinese University of Hong Kong, Hong Kong, China; 8 Department of Psychiatry, The University of Hong Kong, Hong Kong, China; 9 Faculty of Medicine, Jockey Club Centre for Osteoporosis Care and Control, The Chinese University of Hong Kong, Hong Kong, China; 10 School of Public Health, Faculty of Medicine, The Chinese University of Hong Kong, Hong Kong, China; 11 Department of Endocrinology and Metabolism, University Hospital, Reykjavik, Iceland; 12 Faculty of Medicine, University of Iceland, Reykjavík, Iceland; 13 Department of Orthopedic Surgery, Kyungpook National University School of Medicine, Daegu, Republic of Korea; University of Michigan, United States of America

## Abstract

Most genome-wide association (GWA) studies have focused on populations of European ancestry with limited assessment of the influence of the sequence variants on populations of other ethnicities. To determine whether markers that we have recently shown to associate with Bone Mineral Density (BMD) in Europeans also associate with BMD in East-Asians we analysed 50 markers from 23 genomic loci in samples from Korea (*n* = 1,397) and two Chinese Hong Kong sample sets (*n* = 3,869 and *n* = 785). Through this effort we identified fourteen loci that associated with BMD in East-Asian samples using a false discovery rate (FDR) of 0.05; 1p36 (*ZBTB40*, *P* = 4.3×10^−9^), 1p31 (*GPR177*, *P* = 0.00012), 3p22 (*CTNNB1*, *P* = 0.00013), 4q22 (*MEPE*, *P* = 0.0026), 5q14 (*MEF2C*, *P* = 1.3×10^−5^), 6q25 (*ESR1*, *P* = 0.0011), 7p14 (*STARD3NL*, *P* = 0.00025), 7q21 (*FLJ42280*, *P* = 0.00017), 8q24 (*TNFRSF11B*, *P* = 3.4×10^−5^), 11p15 (*SOX6*, *P* = 0.00033), 11q13 (*LRP5*, *P* = 0.0033), 13q14 (*TNFSF11*, *P* = 7.5×10^−5^), 16q24 (*FOXL1*, *P* = 0.0010) and 17q21 (*SOST*, *P* = 0.015). Our study marks an early effort towards the challenge of cataloguing bone density variants shared by many ethnicities by testing BMD variants that have been established in Europeans, in East-Asians.

## Introduction

Geographic ancestry is an important factor when considering the genetic component of the risk of common complex diseases. Variations in the underlying LD structure and in the frequency of sequence variants are observed between populations of different ancestry and are factors that influence the results of association studies. An example of differences in risk assessment of a particular sequence variant between ethinicities is the Atrial Fibrillation (AF) locus on chromosome 4q25 where two variants confer risk of AF in people of European descent. One of these two variants also confers risk in Chinese individuals, where it is observed in higher frequency and with a lower associated risk of AF, than in Europeans [1]. Another example of differences in both frequency and risk of a variant is at a new locus for primary open-angle Glaucoma where a reversed situation is observed, a lower frequency and higher risk in Chinese population compared to Europeans [2]. Furthermore, a recent ancestry-shift refinement effort at the *ESR*1 locus for breast cancer demonstrated that despite apparent lack of association at this locus in Europeans of a variant that was identified in East-Asians an underlying risk variant common to both Europeans and East-Asians could be identified [3].

Bone mineral density (BMD) is the single best predictor of fragility fractures [4], [5] and is used as a reference standard for the description of osteoporosis [6]. It is a highly familial quantitative trait, with heritability estimates in the range of 0.6–0.8 [7], [8], but is also influenced by environmental and medical factors. Genome-wide SNP association (GWA) studies have provided an important opportunity to discover genes with sequence variants contributing to bone density variations, and hence, osteoporosis. Through GWA studies and meta-analysis of GWA studies we, and others, have reported 23 genomic loci that associate with BMD at the genome-wide significance (GWS) level in populations of European ancestry [9]–[12]. Most GWA studies, including those of BMD [9]–[15], have focused on populations of European ancestry. Assessments of the effect of the identified sequence variants have been limited in populations of other ethnicities. Recently, studies in other populations than those of European descent have both yielded new loci and information on their effect across ethnicities [15]–[17], representing an early attempt to assess the effect of these loci globally.

Here, we investigate if the BMD-related genetic variants we previously reported in Europeans also associate with BMD in East-Asians (Korean and two Chinese Hong Kong sample sets). We tested markers from the 23 European GWS loci from our previous scans and a meta-analysis [9], [10], [12].

## Results and Discussion

### Samples, marker selection for genotyping and analysis

The East-Asian samples used in this study comprised of Korean postmenopausal women [18] (*n* = 1,397), Chinese community-dwelling elderly of both sexes from Hong Kong [19] (*n* = 3,869), hereafter called the Hong Kong-I samples, and Southern Chinese females from Hong Kong with extreme BMD phenotypes (*n* = 785) [17], hereafter called the Hong Kong–II samples. Full description of the studies is given in the Methods section and summary characteristics of the study populations are shown in **Table 1**.

**Table 1:** Summary characteristics of the study populations.

	Hong Kong -I		Honk Kong-II		Korea
	Men	Women	Women		Women
			high BMD	low BMD	
#	1882	1984	376	424	1396
Age (SD), year	72.41 (5.02)	72.59 (5.36)	46.6 (14.8)	51.1 (15.9)	59.06 (7.36)
Age-range, year	65–92	65–98	20–84	20–80	45–87
BMI (SD), kg/m2	23.45 (3.12)	23.92 (3.45)	24.58(4.11)	21.14(2.99)	23.4 (2.79)
Height (SD), cm	163.06 (5.76)	150.92 (5.31)	158 (6)	153 (7)	154.99 (5.31)
Weight (SD), kg	62.43 (9.37)	54.52 (8.5)	61.1 (9.9)	49.1 (6.7)	56.21 (7.15)
Hip BMD (SD)	0 (1)	0 (1)	1.15 (0.79)	−1.36 (0.60)	0 (1)
Spine BMD (SD)	−0.02 (0.99)	0 (1)	1.05 (0.78)	−1.59 (0.53)	0 (1)

Mean age, height, weight, and BMI are shown for each study with standard deviations (SD) in parenthesis. Mean of adjusted hip BMD and spine BMD is shown (SD) and the age-range of each study. Hong Kong-II: low BMD subjects are defined as an individual having BMD Z-score ≤−1.28 at either lumbar spine (LS) or femoral neck (FN), which is equivalent to the lowest 10% of the total population, while high BMD subjects are individuals with BMD Z-scores ≥ +1.0 at either of the two skeletal sites.

We tested markers from all 23 European GWS loci that we identified in our previous scans and a meta-analysis [9], [10], [12]. We analysed in the East-Asian samples a total of 50 SNPs (47 GWS and 3 suggestive) from these 23 genomic loci. We directly genotyped these markers in the Korean and the Hong Kong-I samples and performed an in-silico lookup in the Hong Kong-II extreme BMD samples which had previously been genotyped on an Illumina SNP chip.

BMD at the hip (femoral-neck, FN or total-hip) and at the lumbar spine (LS) was adjusted for age and body weight for each sex and population separately. For each SNP, the allele that associated with lowered BMD in the European populations was tested separately for its association with BMD of the hip and for its association with BMD of the spine. The results for the three East-Asian sample sets were combined using inverse variance weighted Z-scores.

The Simes procedure was used to control the FDR [20] in the overall analysis rather than the conservative Bonferroni adjustment, due to the large number of true positives that we expected to find. We chose to use a FDR threshold of 0.05 which would have corresponded to using a P value threshold of 0.001 based on the Bonferroni adjustment, while the Simes procedure resulted in P values below 0.021 and 0.025 being declared to associate with spine and hip BMD, respectively. The Simes procedure was also used to control the FDR when testing difference in effect between the two ethnicities, resulting in a threshold of 0.012 and 0.008 for spine and hip BMD, respectively.

### Investigation of European bone density loci in East-Asians

The previously reported GWS loci in European populations that we tested were 1p36 (*ZBTB40*), 1p31 (*GPR177*), 2p21 (*SPTBN1*), 3p22 (*CTNNB1*), 4q22 (*MEPE*), 5q14 (*MEF2C*), 6p21 (MHC), 6q25 (*ESR1*), 7p14 (*STARD3NL*), 7q21 (*FLJ42280*), 8q24 (*TNFRSF11B*), 11p15 (*SOX6*), 11p11 (*ARHGAP1*) 11p13 (*DCDC5*), 11q13 (*LRP5*), 12q13 (*SP7*), 13q14 (*TNFSF11*), 14q32 (*MARK3*), 16q24 (*FOXL1*), 17q21 (*SOST*), 17q21 (*HDAC5*), 17q12 (*CRHR1*), and 18q21 (*TNFRSF11A*) [9], [10], [12]. The full set of markers tested is shown in **Table S1**.

Thirty markers from fourteen loci associated with BMD in East-Asians in this study (FDR<0.05). Markers at ten loci associated with both hip and spine BMD: 1p36, 1p31, 3p22, 4q22, 6q25, 7p14, 7q21, 8q24, 11q13 and 13q14. No other loci associated with spine BMD while four additional loci associated with hip BMD: 5q14, 11p15, 16q24, 17q21. The results for the markers associating with spine and hip BMD in East-Asians are shown in **Table 2** and **Table 3**, respectively, and the results for all the tested markers are shown in **Table S2** and **Table S3**.

**Table 2:** Association of European loci in East-Asian samples; spine BMD.

					Korea (n = 1,396)		HongKong-I (n = 3,736)		Hong Kong-II (n = 785)		(n = 5,917)			
Locus	SNP	Allele	Freq. Europe	Freq. Asia	Effect	P value	Effect	P value	Effect	P value	Effect Asia	*P* value Asia	P_het_	*I* ^2^
1p36	rs7524102	A	0.830	0.794	−0.09	0.041	−0.08	0.0034	−0.07	0.015	−0.08	5.5e-05	0.96	0.0
	rs6696981	G	0.864	0.803	−0.08	0.065	−0.08	0.0024	−0.12	0.0011	−0.09	7.0e-06	0.68	0.0
	rs6426749	G	0.830	0.798	−0.08	0.045	−0.09	0.00070	−0.08	0.0080	−0.09	7.5e-06	0.96	0.0
	rs7543680	G	0.772	0.763	−0.07	0.075	−0.06	0.0085	−0.07	0.028	−0.07	0.00036	0.99	0.0
1p31	rs2566755	A	0.790	0.773	−0.14	0.0010	−0.06	0.021	−0.06	0.045	−0.07	0.00012	0.23	32.8
3p22	rs10490823	G	0.460	0.742	−0.07	0.055	−0.05	0.035	−0.12	0.00095	−0.07	0.00015	0.32	12.0
4q22	rs1471403	C	0.660	0.665	−0.05	0.11	−0.05	0.016	−0.04	0.085	−0.05	0.0026	0.93	0.0
6q25	rs7751941	A	0.217	0.011	−0.51	0.0075	−0.17	0.055	−0.22	0.050	−0.23	0.0014	0.35	4.9
7p14	rs1524058	T	0.400	0.432	−0.04	0.14	−0.06	0.0028	−0.05	0.029	−0.06	0.00025	0.87	0.0
7q21	rs4729260	G	0.320	0.134	−0.03	0.33	−0.01	0.35	−0.16	3.0e-05	−0.07	0.0027	0.013	77.0
	rs7781370	T	0.340	0.132	−0.06	0.16	−0.04	0.12	−0.16	3.4e-05	−0.08	0.00017	0.066	63.2
8q24	rs4355801	A	0.536	0.766	−0.02	0.35	−0.09	0.00080	−0.08	0.030	−0.07	0.00037	0.33	9.4
	rs2062377	T	0.560	0.339	−0.02	0.30	−0.08	0.0022	−0.06	0.033	−0.06	0.00065	0.50	0.0
	rs6469792	C	0.529	0.619	−0.00	0.52	−0.08	0.00055	−0.10	0.0019	−0.07	3.4e-05	0.21	35.5
	rs6469804	A	0.518	0.805	−0.01	0.40	−0.08	0.0029	−0.10	0.0080	−0.07	0.00048	0.28	20.9
	rs6993813	C	0.504	0.660	−0.00	0.52	−0.07	0.0043	−0.09	0.0037	−0.06	0.00032	0.30	17.1
11q13	rs599083	G	0.310	0.264	−0.11	0.0029	−0.04	0.070	−0.04	0.10	−0.06	0.0014	0.30	16.8
13q14	rs7992970	A	0.777	0.679	−0.03	0.23	−0.07	0.0029	-0.10	0.0026	−0.07	7.5e-05	0.45	0.0
	rs9594738	T	0.568	0.086	−0.14	0.0095	−0.07	0.055	−0.09	0.060	−0.09	0.0010	0.59	0.0
	rs10507508	A	0.947	0.876	−0.03	0.34	−0.09	0.0023	−0.13	0.0036	−0.09	0.00011	0.42	0.0
	rs9594751	T	0.265	0.065	−0.14	0.021	−0.12	0.0085	−0.10	0.070	−0.12	0.0003	0.92	0.0

The SNPs that associated with spine BMD in the East-Asian samples in this study are shown. The SNP alleles that associate with lower BMD in Europeans are shown and their frequency in Europeans and East-Asians. The estimated effect on lumbar spine BMD is expressed as standard deviations below the mean. Single-sided P values are shown for the individual sample sets separately (Korea, Hong Kong-I, Hong Kong-II) and the three samples sets combined. Heterogeneity P values (P_het_) and estimates (*I* ^2^) are given.

**Table 3:** Association of European loci in East-Asian samples; hip BMD.

					Korea (n = 1,396)		HongKong-I (n = 3,736)		Hong Kong-II (n = 785)		(n = 5,988)			
Locus	SNP	Allele	Freq. Europe	Freq. Asia	Effect	P value	Effect	P value	Effect	P value	Effect Asia	P value Asia	P_het_	*I* ^2^
1p36	rs7524102	A	0.830	0.794	−0.11	0.012	−0.11	8.0e-05	−0.09	0.0014	−0.10	5.5e-08	0.94	0.0
	rs6696981	G	0.864	0.803	−0.13	0.0070	−0.10	0.00016	−0.15	9.0e-05	−0.12	8.0e-09	0.59	0.0
	rs6426749	G	0.830	0.798	−0.12	0.0060	−0.12	1.5e-05	−0.10	0.00090	−0.11	4.3e-09	0.86	0.0
	rs7543680	G	0.772	0.763	−0.08	0.034	−0.08	0.0023	−0.09	0.012	−0.08	2.5e-05	0.97	0.0
1p31	rs2566755	A	0.790	0.773	−0.15	0.00075	−0.05	0.029	−0.06	0.041	−0.07	0.00014	0.17	42.7
3p22	rs10490823	G	0.460	0.742	−0.07	0.055	−0.06	0.0090	−0.09	0.0085	−0.07	0.00013	0.80	0.0
	rs87938	A	0.450	0.639	−0.06	0.070	−0.04	0.065	−0.03	0.14	−0.04	0.0095	0.85	0.0
4q22	rs1471403	C	0.660	0.665	0.00	0.50	−0.06	0.0060	−0.03	0.18	−0.05	0.0065	0.63	0.0
5q14	rs1366594	C	0.450	0.584	−0.08	0.022	−0.05	0.028	−0.09	0.00032	−0.07	1.3e-05	0.41	0.0
6q25	rs9478223	C	0.104	0.053	−0.11	0.10	−0.16	0.0031	−0.00	0.52	−0.09	0.0070	0.21	36.1
	rs7751941	A	0.217	0.011	−0.29	0.080	−0.06	0.28	−0.37	0.0043	−0.19	0.0075	0.19	39.7
	rs2941740	T	0.570	0.884	−0.11	0.029	−0.07	0.034	−0.07	0.046	−0.08	0.0011	0.81	0.0
	rs1999805	C	0.440	0.754	−0.01	0.42	−0.08	0.0014	−0.02	0.27	−0.05	0.0047	0.28	22.1
	rs2504063	A	0.400	0.806	−0.05	0.15	−0.08	0.026	−0.04	0.14	−0.05	0.011	0.73	0.0
7p14	rs1524058	T	0.400	0.432	−0.04	0.14	−0.07	0.0015	−0.02	0.26	−0.05	0.0020	0.37	0.0
7q21	rs4729260	G	0.320	0.137	−0.02	0.37	−0.03	0.16	−0.17	9.0e-06	−0.08	0.00038	0.017	75.6
	rs7781370	T	0.340	0.132	−0.03	0.32	−0.04	0.12	−0.16	1.9e-05	−0.08	0.00025	0.039	69.1
8q24	rs6469792	C	0.529	0.619	0.05	0.89	−0.06	0.0085	−0.13	8.5e-05	−0.05	0.00095	0.0029	82.9
	rs6993813	C	0.504	0.655	0.03	0.80	−0.04	0.050	−0.12	0.00030	−0.05	0.0055	0.013	77.1
11p15	rs7117858	A	0.800	0.791	−0.08	0.028	−0.04	0.10	−0.10	0.0018	−0.07	0.00033	0.34	6.8
11q13	rs599083	G	0.310	0.264	−0.05	0.12	−0.03	0.12	−0.07	0.0085	−0.05	0.0033	0.59	0.0
13q14	rs7992970	A	0.777	0.679	−0.06	0.070	−0.04	0.050	−0.06	0.055	−0.05	0.0037	0.86	0.0
	rs10507508	A	0.947	0.876	−0.15	0.0080	−0.04	0.090	−0.11	0.011	−0.08	0.00075	0.25	28.9
16q24	rs10048146	G	0.190	0.288	−0.09	0.011	−0.07	0.0055	−0.02	0.27	−0.05	0.0010	0.26	26.6
17q21	rs1513670	A	0.371	0.595	−0.01	0.35	−0.05	0.021	−0.03	0.16	−0.04	0.015	0.75	0.0

The SNPs that were associated with hip BMD in the East-Asian samples in this study are shown. The SNP alleles that associate with lower BMD in Europeans are shown and their frequency in Europeans and East-Asians. The estimated effect on hip BMD is expressed as standard deviations below the mean. Single-sided *P* values are shown for the individual sample sets separately (Korea, Hong Kong-I, Hong Kong-II) and the three sample sets combined. Heterogeneity P values (P_het_) and estimates (*I* ^2^) are given.

### Loci associated with both spine and hip BMD in East-Asian samples

The 1p36 locus associated most significantly with BMD in this study, typified by rs6426749 with a *P* = 4.3×10^−9^ for hip BMD and 7.5×10^−6^ for spine BMD. The estimated effect in the East-Asians was very similar to that in the Europeans; rs6426749 associated with 0.11 SD lower hip BMD and 0.09 lower spine BMD per copy of the G allele in the East-Asians compared to 0.08 and 0.11 SD in the Europeans, for hip BMD and spine BMD, respectively [12]. All four markers tested at this locus are of very similar frequencies in both ethnic groups (around 80% allelic frequency), are highly correlated and are therefore assumed to capture the same association signal.

Rs2566755 at the second locus on chromosome 1, 1p31, also associated with both hip BMD and spine BMD in the East-Asian samples with *P* = 0.00014 for hip-BMD and *P* = 0.00012 for spine-BMD, and similar effect as in Europeans. The frequency of this SNP is also comparable in both ethnic groups; the allele that associates with lower BMD [A] is very frequent, between 77 and 79 percent.

We tested two markers at the 3p22 (*CTNNB1*) locus; rs87938 derived from the meta-analysis results and rs10490823, which we had followed in our previous GWA study but had not reached GWS [10]. The rs10490823 SNP associated with both hip BMD (*P* = 0.00013) and spine BMD (*P* = 0.00015) in the East-Asians and rs87938 associated with hip BMD (*P* = 0.0095). Again, the effect was very similar in the two ethnic groups; the rs10490823-G allele associated with 0.07 SD lower hip BMD in East-Asians compared to 0.06 SD in Europeans. The frequency of associated allele is again high in both ethnicities, 46% in Europeans and 73% in the East-Asians. These two markers, rs10490823 and rs87938, are fully equivalent in the HapMap-CEU samples (r^2^ = 1) and substantially correlated but not equivalent (r^2^ = 0.57, D' = 1) in the HapMap-JPT+CHB dataset. They are located 14 kb apart, about 100 kb upstream of the *CTNNB1* (catenin (cadherin-associated protein), beta 1) gene.

The SNP rs1471403-C at 4q22 (*MEPE*) associated with both hip BMD (*P* = 0.0065) and spine BMD (*P* = 0.0026) in the East-Asians. Frequency of the rs1471403 C allele is comparable in Europe and in the East-Asian samples; 65–70%, and the effect is similar in both groups, the C allele associated with 0.05 SD lower hip BMD and spine BMD.

The 6q25 (*ESR1*) is an example of a complex locus where most markers differ in frequencies between Europeans and East-Asians; only three of the ten markers that we tested are comparable in frequency in the two ethnic groups. This locus also showed a complex pattern of association in our previous study of the European samples; the association signal could not be explained by a single SNP and at least three SNPs were needed to fully capture the association [9]. Five markers associated with hip BMD and one markers with spine BMD in the East-Asians; the rs7751941 (allele A, 1.1% freq.) in common to both skeletal sites, *P* = 0.0075 for hip BMD and *P* = 0.0014 for spine BMD. These data underscore the highly complex architecture of this locus in association with BMD, in line with our recent study examining this same locus for the risk of breast cancer across ethnicities [3]. A similar study of ancestry-shift refinement mapping of this locus with regards to BMD is warranted in order to pinpoint the underlying signal.

The marker rs1524058-T, at 7p14 (*STARD3NL*), associated with both spine BMD (*P* = 0.00025) and hip BMD (*P* = 0.0020) in the East-Asians. A recent GWA study in East-Asians identified rs1721400 at this same locus as associated with BMD of the radius, tibia and heel [16]. That marker did not associate with BMD in Europeans [12] whereas rs1524058 associated with BMD in people of East-Asian ancestry in this study as well as in Europeans in the previous meta-analysis. Rs1721400 and rs1524058 are located 75 kb apart and are somewhat correlated in the HapMap-JPT+CHB dataset (r^2^ = 0.230, D' = 0.931), whereas limited linkage-disequilibrium (LD) is observed for the CEU data (r^2^ = 0.015, D' = 0.194). This suggests that the rs1524058 is perhaps closer to the underlying signal at this locus than rs1721400.

At the second chromosome 7 locus, 7q21 (*FLJ42280*), both markers associated with both hip BMD and spine BMD. These markers showed significant heterogeneity between the studies, explained by the large effect on BMD in the Hong Kong-II samples.

All five markers at the 8q24 (*TNFRSF11B*, OPG) locus associated with spine BMD in the East-Asians, rs6469792-C most significantly (*P* = 3.4×10^−5^), and two of the five, rs6469792-C and rs6993813-C, also associated with hip BMD. Substantial heterogeneity was observed for markers at this locus for hip BMD. This locus was more strongly associated with spine BMD than hip BMD in previous studies of Europeans, although it was GWS for both skeletal sites.

The 11q13 (*LRP5*) rs599083-G marker associated with both spine BMD (*P* = 0.0014) and hip BMD (*P* = 0.0033) in the East-Asians. Many studies have previously shown association between markers in the *LRP5* (low density lipoprotein receptor-related protein 5) gene and BMD, both in East-Asians [21]–[25] and in Europeans, although these have only reached GWS in Europeans [11], [12], [26].

We analysed seven markers at the 13q14 (*TNFSF11*) locus. The frequency of five of these markers is markedly different in the East-Asian populations from that of the Europeans. This locus was the most strongly associated spine BMD locus in our previous European studies, represented by T-rs9594738 [9], [10] and T- rs9533090 [12]. Both markers associated nominally with spine BMD in the East-Asian samples, and rs9594738 associated with spine BMD (*P* = 0.0010). The frequency of these SNPs is very different in Asia from that in Europe, 8–9% in East-Asia, versus 50–56% in Europe. Three other markers, rs9594751-T, rs7992970-A and rs10507508-A, varying in frequency from 6%–68%–89%, respectively, in the East-Asians, associated with spine BMD (*P* = 0.00030, 7.5×10^−5^, 0.00011, respectively). The rs7992970-A and rs10507508-A markers also associated with hip BMD (*P* = 0.0037, 0.00075, respectively).

### Loci that associated with hip BMD in East-Asian samples

The two loci, 5q14 (*MEF2C*) and 11p15 (*SOX6*), showed highly significant skeletal site specificity in the European meta-analysis [12], only presenting significant association with hip BMD and not with spine BMD. The same pattern of skeletal site specificity was observed in the East-Asian samples, association was observed between markers at both loci and hip BMD but not for spine BMD; rs1366594-C at 5q14 and rs7117858-A at 11p15 associated with hip BMD with *P* = 1.3×10^−5^ and *P* = 0.00033, respectively.

The marker rs10048146 at 16q24 (*FOXL1*) was associated with both hip BMD and spine BMD in the meta-analysis of European studies [12], but reached GWS level only for spine BMD. In this study of East-Asians rs10048146-G, however, only associated with hip BMD, *P* = 0.0010, and although a nominal association was observed for spine BMD, *P = *0.040. The effect on spine BMD is, however, not significantly different between East-Asians and Europeans (**Table S4**).

Rs1513670-A, one of three markers tested at the 17q21 (*SOST*) locus, was associated with hip BMD in the East-Asian samples (*P* = 0.015) but not with spine BMD. Markers at this locus did not reach GWS in the meta-analysis of European samples but were significantly associated with total hip BMD and not with spine BMD in our previous study [10]. In the same study we showed, by conditional analysis, that there were probably two independent signals at this locus, one of which was captured by rs1513670.

### Effect estimates in East-Asians versus Europeans

We compared the estimated effect of the SNP alleles on BMD in the East-Asians with the estimated effect of the SNPs in Europeans (**Table S4**). Of the fifty markers tested there were fourteen markers that showed significant differences in effect between East-Asians and Europeans; twelve for spine BMD and eight for hip BMD, six common to both sites. Five of the shared markers are located in the complex 6q25 locus (rs9479055, rs4870044, rs1038304, rs6929137, rs6900157) and one marker at 13q14 (rs9594759). For hip BMD the 11p11 (rs7932354) and 18q21 (rs3018362) also showed significant difference in effect, whereas for spine BMD markers at 6p21 (rs3130340), 11p15 (rs7117858), 11p13 (rs16921914) and 17q21 (rs9303521) showed difference in effect. Other markers at the 6q25 and 13q14 loci did not differ in effect, leaving six loci signficantly different in their effect on BMD between East-Asians and Europeans using the set of markers tested here. The lack of association of the 2p21, 14q32 and 17q21 (*HDAC5*) loci seems due to lack of power in this study rather than true differences in the association with BMD between Europeans and East-Asians, whereas the effect of the markers used in this study at 6p21, 11p13, 11p11 and 17q21 (*CRHR1*) are likely to reflect true differences between the ethnic groups.

### Conclusion

Here, we report the influence of sequence variants that were discovered in European populations on BMD in East-Asians, with successful identification of fourteen loci associating with BMD. The effect of BMD association in each of these loci is very similar to that observed in the European samples; the alleles that associated with low BMD associated with similar BMD decrease in SD in both ethnicities. Furthermore, the frequency of the associated allele is similar in both ethnicities for the majority, but not all, of the associating markers as is evident for the complex 6q25 and 13q14 loci. We chose to use the FDR test [20] to determine association because of the a-priori expectation of true associations. The more conservative Bonferroni correction of the *P* values, accounting for all markers tested in this study (*P*<0.05/50), would consider ten of the fourteen loci significant (1p36, 1p31, 3p22, 5q14, 7p14, 7q21, 8q24, 11p15, 13q14 and 16q24) and sixteen loci nominally signficant.

The results for other European loci were inconclusive, although overall, the effect sizes observed in East-Asians are similar to those in Europeans and only differ significantly for fourteen markers in total (twelve for spine and eight for spine, six in common to both sites). The non-associating loci perhaps reflect ancestry-specific loci or that the European markers tested here are not capturing the true association signals due to ethnic LD differences. These data also indicate that further refinement across different ethnicities, which was not undertaken in this study, may aid in identifying the sequence variants that truly affect BMD. Such ancestry-shift refinement mapping of each of the loci would enable clarification of the observed signals and determination of ancestry-specificity or the potential global effect of these loci on BMD.

## Materials and Methods

### Ethics Statement

All participants provided written informed consent. The study was approved by Data Protection Commission of Iceland (DPC), the National Bioethics Committee of Iceland, the AMC Ethics Review Committee (Seoul), the Clinical Research Ethics Committee of the Chinese University of Hong Kong local institutional review boards of University of Hong Kong.

### Study Populations

The East-Asian samples used for this study were from Hong Kong and Korea.

#### Hong Kong-I: Chinese community elderly Men study and the Chinese community elderly Women study

Two sets of samples of different sex, each including 2000 Chinese subjects living in the community, aged 65 years and above, were recruited by posting public advertisements at housing estates and community centers for the elderly in Hong Kong since 2001. To ensure an even distribution of age, stratified sampling technique was used where approximately one-thirds of participants were selected from each of the following age groups: 65–69, 70–74, and  = 75 years old. All subjects were invited to the School of Public Health of the Chinese University of Hong Kong to have their peripheral blood samples collected for DNA extraction, and measurements of their body weight (kg), height (cm), body mass index (BMI) and bone mineral density (BMD) at various body sites were also taken. In addition, a questionnaire was administered to collect information regarding their age, smoking habit, and for women subjects specifically, their history of pregnancy and breast feeding, age of menarche and menopause, and history of estrogen treatment. The men cohort contributed to the international Mr. Os study of osteoporosis in men [27]. Baseline BMD measurements: BMD at the lumbar spine (LS, L1–L4), femoral neck (FN), total hip (TH), and whole body (WB) were measured by a dual energy X-ray absorptiometry (DXA) (Hologic QDR-4500W Densitometer, Hologic Delphi, Hologic Inc, Bedford, MA). The in vivo precision of DXA was 0.9% at the lumbar spine, 0.7% at the hip, and 1.04% for whole body [27]. BMD normative reference for the Hong Kong Chinese population was reported previously [19]. All participants provided informed consent. The study was approved by the Clinical Research Ethics Committee of the Chinese University of Hong Kong.

#### Hong Kong-II: subjects with extreme BMD

The samples of 785 unrelated women with extreme high or low BMD were selected from HKSC samples with extreme BMD (n = 1,520) and served as a discovery dataset in [17]. These subjects were selected from a database (>7,000 Southern Han Chinese volunteers) of the Osteoporosis Centre of the University of Hong Kong. The low BMD subjects are defined as an individual having BMD Z-score ≤−1.28 at either lumbar spine (LS) or femoral neck (FN) (the lowest 10% of the total populations), while high BMD subjects are individuals with BMD Z-score ≥ +1.0 at either sites. Subjects that were reported to have diseases or environmental factors that may affect BMD and bone metabolism were excluded. The recruitment procedure and exclusion criteria have been detailed elsewhere [28]. BMD (g/cm2) at LS and FN was measured by dual-energy X-ray absorptiometry (DXA; Hologic QDR 4500 plus, Hologic Waltham, MA, USA) with standard protocol. The age corrected and standardized BMD (mean 0, SD 1), termed BMD Z-scores was generated. Selecting extreme valued individuals for genotyping is known to inflate effect estimates. In order to estimate this inflation factor, we simulated two large normal populations each with standard deviation 1, but one having mean 0 and the other having mean 0.01. We then applied extreme value thresholding until we observed phenotype variance like that of the selected samples and used the inflation of the estimated difference in mean between the two simulated populations to estimate the BMD effect inflation. For spine BMD the observed inflation in variance was 2.14 and the effects were estimated to be inflated by a factor of 2.24. Similarly for hip BMD the observed inflation in variance was 2.06 and the effects were estimated to be inflated by a factor of 2.14. We therefore deflated all effect estimates based on the Hong Kong-II sample by these factors. The in vivo precision of the machine was 1.2% and 1.5% for LS and FN BMD, respectively [29]. All participants provided informed consent and the study protocols were reviewed and approved by local institutional review boards.

#### Korea

The study population comprised 1,408 postmenopausal women of Korean ethnicity who visited the Osteoporosis Clinic of Asan Medical Center (AMC, Seoul, Korea) [18]. Menopause was defined as the absence of menstruation for at least 6 months and confirmed by measurement of serum follicle-stimulating hormone (FSH). Women who were prematurely menopausal (<40 years of age) were excluded. Women who had taken drugs that might affect bone metabolism for >6 months or within the previous 12 months were also excluded. Subjects were excluded if they had suffered from any disease that might affect bone metabolism. Women who had had a stroke or dementia were also excluded because of concerns related to their limited physical activity. Women were also excluded if they had osteophyte formation above the fourth grade of the Nathan classification and/or severe facet joint osteoarthritis in the lumbar spine using conventional spine radiographs. Areal BMD (g/cm2) was measured at the proximal femur and the anterior-posterior lumbar spine (L1 to L4) using dual energy X-ray absorptiometries (DXAs) (Expert XL, Lunar, Madison, WI, USA; Prodigy Advance, GE, Madison, WI, USA; and QDR 4500-A, Hologic, Waltham, MA, USA). Short-term in vivo measurement precision for the Lunar Expert, Lunar Prodigy and Hologic machines, expressed as the coefficient of variation, were 0.82%, 0.67% and 0.85% for the lumbar spine, respectively, and 1.12%, 1.25% and 1.20% for the femoral neck, respectively. Cross-calibration equations among the three systems were derived in 109 healthy Korean women (55±11 years, range 31–75 years), and were calculated as follows; Lumbar BMD (g/cm2): Lunar Expert  = 1.0373× Lunar Prodigy −0.0353 Lunar Expert  = 1.1287× Hologic −0.0027; Femur neck BMD (g/cm^2^): Lunar Expert  = 1.0579× Lunar Prodigy −0.0275; Lunar Expert  = 1.1556× Hologic +0.0182. All participants provided informed consent. The study was approved by the AMC Ethics Review Committee (Seoul).

### Genotyping

The Hong Kong-I and the Korean samples were genotyped by single-SNP Centaurus (Nanogen) assays at deCODE Genetics [30]. The quality of each Centaurus SNP assay was evaluated by genotyping each assay in the CEU HapMap samples and comparing the results with the HapMap data. Assays with a mismatch rate >1.5% were not used, and a linkage disequilibrium (LD) test was used for markers known to be in LD. The Hong Kong-II samples were genotyped using the Infinium assay (Illumina, San Diego, USA) with Human610-quad chip. 785 individuals were retained for analysis after exclusion based on strict quality-control criteria; 1) genotyping call rate less than 95%, 2) autosomal heterozygosity less than 27% or more than 31%, 3) being related or identical to other individuals in the sample and 4) discordance of observed gender and estimated gender. All SNPs used in this study satisfied following criteria: 1) genotyping call rate of 95%, 2) Hardy–Weinberg equilibrium (HWE) p value above 1.0×10^−4^, 3) minor allele frequency (MAF) more than 0.01.

### Standardization and Association analysis

For the Korean and Hong Kong –I studies, age and weight corrected bone mineral density (BMD) was computed for each sex and population separately to have a mean 0 and standard deviation 1. For each SNP, a linear regression, using the genotype as an additive covariate and standardized BMD as the response, was fitted to test for association. For each SNP, the allele that associated with lowered BMD in European populations was tested separately for its association with BMD of the hip and for its association with BMD of the lumbar spine. Single-sided *P* values are reported for the tests for associations in the East-Asians.

An overall Z -score was calculated by using inverse variance weighted Z -scores. An overall estimate of the effect per allele was calculated by weighting together the effects in each population by the population's effective sample size.

### Heterogeneity analysis

Heterogeneity is tested by comparing the null hypothesis of the effect being the same in all populations to the alternative hypothesis of each population having a different effect using a likelihood ratio test. *I* ^2^ lies between 0% and 100% and describes the proportion of total variation in study estimates that is due to heterogeneity [31].

## Supporting Information

Table S1 Markers tested in this study. All SNPs analysed in the East-Asian samples are shown, their chromosomal position, alleles and the tested allele. The frequency of the allele that associated with lowered BMD in Europeans is shown and its effect on spine BMD and hip BMD along with the respective P values.
(0.24 MB DOC)

Table S2 Results for spine BMD for all the SNPs tested in the East-Asian samples. The effect on spine BMD in the East-Asian populations of all SNPs tested in this study. The frequency of the allele that associated with lowered BMD in Europeans is shown along with its effect on spine BMD. A FDR of 0.05, corresponding to a P value threshold of 0.021, was used to determine significance of association.
(0.16 MB DOC)

Table S3 Results for hip BMD for all the SNPs tested in the East-Asian samples. The effect on hip BMD in the East-Asian populations of all SNPs tested in this study. The frequency of the allele that associated with lowered BMD in Europeans is shown along with its effect on hip BMD. A FDR of 0.05, corresponding to a P value threshold of 0.025, was used to determine significance of association.
(0.16 MB DOC)

Table S4 Difference in effect on BMD between Europeans and East-Asians. The effect on hip BMD and spine BMD in populations of European descent and of East-Asian descent of SNPs tested in this study. The frequency of the allele that associated with lowered BMD in Europeans is shown for Europeans and for East-Asians along with its effect on spine BMD and hip BMD in both ethnicities. The P value comparing the effect between the two ethnicities is shown. Effects deemed different using a FDR of 0.05 corresponds to a P value threshold of 0.012 for Spine BMD and 0.008 for Hip BMD.
(0.13 MB DOC)
