# Supplementary material for: European Bone Mineral Density Loci Are Also Associated with BMD in East-Asian Populations
Source: PLoS One. 2010 Oct 7;5(10):e13217. doi: 10.1371/journal.pone.0013217 (PMC2951352; doi:10.1371/journal.pone.0013217)
Supplement: Table S1 — Markers tested in this study. All SNPs analysed in the East-Asian samples are shown, their chromosomal position, alleles and the tested allele. The frequency of the allele that associated with lowered BMD in Europeans is shown and its effect on spine BMD and hip BMD along with the respective P values. (0.24 MB DOC) [file pone.0013217.s001.doc]

**Table S1. Markers tested in this study**

All SNPs analysed in the East-Asian samples are shown, their chromosomal position, alleles and the tested allele. The frequency of the allele that associated with lowered BMD in Europeans is shown and its effect on spine BMD and hip BMD along with the respective *P* values.

| **Locus** | **SNP** | **Ref.** | **Chr** | **build36** | **alleles** | **allele** | **Freq.** | **Effect spine** | ***P* value spine** | **Effect hip** | ***P* value hip** |
| --- | --- | --- | --- | --- | --- | --- | --- | --- | --- | --- | --- |
| 1p36 | rs7524102 | [1,2] | 1 | 22571034 | A/G | A | 0.830 | -0.11 | 7.0E-09 | -0.15 | 1.2E-16 |
|  | rs6696981 | [1] | 1 | 22575445 | G/T | G | 0.864 | -0.12 | 2.4E-08 | -0.14 | 2.5E-12 |
|  | rs6426749 | [2] | 1 | 22584060 | C/G | G | 0.830 | -0.11 | 7.6E-10 | -0.08 | 4.8E-08 |
|  | rs7543680 | [3] | 1 | 22603856 | A/G | G | 0.772 | -0.07 | 9.3E-06 | -0.10 | 1.0E-10 |
| 1p31 | rs2566755 | [2] | 1 | 68407978 | A/G | A | 0.790 | -0.10 | 3.3E-13 | -0.10 | 1.7E-12 |
| 2p21 | rs11898505 | [2,3] | 2 | 54538061 | A/G | G | 0.660 | -0.07 | 1.6E-08 | -0.03 | 0.020 |
| 3p22 | rs10490823 | [3] | 3 | 41098739 | A/G | G | 0.460 | -0.05 | 2.8E-04 | -0.06 | 9.4E-06 |
|  | rs87938 | [2] | 3 | 41112676 | A/G | A | 0.450 | −0.04 | 1.7 E-04 | −0.07 | 8.1E-10 |
| 4q22 | rs1471403 | [2] | 4 | 88994267 | C/T | C | 0.660 | -0.07 | 1.5E-08 | -0.06 | 7.8 E-07 |
| 5q14 | rs1366594 | [2] | 5 | 88411817 | G/T | C | 0.450 | 0.01 | 0.65 | −0.09 | 1.3E-13 |
| 6p21 | rs3130340 | [1] | 6 | 32352605 | C/T | T | 0.795 | -0.10 | 1.2E-07 | -0.05 | 0.0065 |
| 6q25 | rs9479055 | [1] | 6 | 151889660 | A/C | C | 0.355 | -0.08 | 6.2E-07 | -0.08 | 3.1E-08 |
|  | rs9478223 | [1] | 6 | 151941931 | C/T | C | 0.104 | -0.14 | 2.5E-10 | -0.11 | 1.1E-06 |
|  | rs4870044 | [1] | 6 | 151943102 | C/T | T | 0.286 | -0.11 | 6.6E-13 | -0.09 | 8.0E-10 |
|  | rs1038304 | [1] | 6 | 151974868 | A/G | G | 0.472 | -0.09 | 1.9E-10 | -0.08 | 4.0E-10 |
|  | rs6929137 | [1] | 6 | 151978370 | A/G | A | 0.299 | -0.10 | 1.6E-10 | -0.08 | 1.5E-08 |
|  | rs7751941 | [3] | 6 | 151988351 | A/G | A | 0.217 | -0.09 | 5.7E-08 | -0.09 | 1.3E-07 |
|  | rs6900157 | [3] | 6 | 151995820 | C/T | C | 0.316 | -0.09 | 4.2E-09 | -0.09 | 2.2E-09 |
|  | rs2941740 | [2] | 6 | 152051331 | C/T | T | 0.570 | -0.07 | 2.0E-09 | -0.07 | 2.0E-10 |
|  | rs1999805 | [1] | 6 | 152110057 | C/T | C | 0.440 | -0.09 | 2.2E-08 | -0.06 | 1.2E-04 |
|  | rs2504063 | [1] | 6 | 152132400 | A/G | A | 0.400 | -0.08 | 1.5E-08 | -0.06 | 4.4E-06 |
| 7p14 | rs1524058 | [2] | 7 | 38102802 | C/T | T | 0.400 | −0.07 | 1.1E-09 | −0.04 | 8.9E-04 |
| 7q21 | rs4729260 | [2] | 7 | 95955854 | C/G | G | 0.320 | −0.08 | 1.7E-10 | −0.09 | 9.4E-12 |
|  | rs7781370 | [2] | 7 | 95971467 | C/T | T | 0.340 | −0.07 | 1.1E-09 | −0.08 | 4.7E-12 |
| 8q24 | rs4355801 | [3] | 8 | 119993054 | A/G | A | 0.536 | -0.09 | 5.2E-10 | -0.07 | 5.3E-07 |
|  | rs2062377 | [2] | 8 | 120076601 | A/T | T | 0.560 | -0.09 | 3.5E-16 | -0.06 | 5.4E-08 |
|  | rs6469792 | [3] | 8 | 120077552 | C/T | C | 0.529 | -0.11 | 1.1E-15 | -0.10 | 3.1E-13 |
|  | rs6469804 | [1] | 8 | 120114010 | A/G | A | 0.518 | -0.12 | 7.4E-15 | -0.08 | 2.5E-09 |
|  | rs6993813 | [1] | 8 | 120121419 | C/T | C | 0.504 | -0.12 | 1.8E-14 | -0.09 | 3.3E-11 |
| 11p15 | rs7117858 | [2] | 11 | 15651038 | A/G | A | 0.800 | 0.04 | 0.0040 | -0.09 | 6.4E-09 |
| 11p13 | rs16921914 | [2] | 11 | 31167347 | A/G | G | 0.730 | -0.08 | 2.3E-09 | -0.04 | 0.0030 |
| 11p11 | rs7932354 | [2] | 11 | 46678797 | C/T | C | 0.710 | -0.06 | 1.1E-05 | -0.07 | 4.0E-09 |
| 11q13 | rs599083 | [2] | 11 | 67948922 | G/T | G | 0.310 | −0.07 | 4.7E-08 | −0.05 | 9.7E-05 |
| 12q13 | rs2016266 | [3] | 12 | 52014222 | A/G | A | 0.680 | -0.07 | 5.2E-08 | -0.05 | 3.0E-04 |
| 13q14 | rs7992970 | [1] | 13 | 41843463 | A/G | A | 0.777 | -0.11 | 2.7E-10 | -0.08 | 8.2E-06 |
|  | rs9533090 | [2] | 13 | 41849449 | C/T | T | 0.500 | −0.12 | 5.4E-25 | −0.041 | 3.9E-04 |
|  | rs9594738 | [1] | 13 | 41850145 | C/T | T | 0.568 | -0.17 | 2.0E-21 | -0.10 | 1.9E-08 |
|  | rs9533093 | [3] | 13 | 41859597 | C/T | T | 0.808 | -0.11 | 5.4E-11 | -0.04 | 0.038 |
|  | rs10507508 | [1] | 13 | 41867782 | A/G | A | 0.947 | -0.17 | 3.6E-07 | -0.13 | 5.3E-05 |
|  | rs9594751 |  | 13 | 41895267 | C/T | T | 0.265 | -0.08 | 3.7E-07 | -0.07 | 2.1E-05 |
|  | rs9594759 | [1] | 13 | 41930593 | C/T | T | 0.622 | -0.13 | 1.1E-16 | -0.07 | 2.1E-06 |
| 14q32 | rs2010281 | [3] | 14 | 102932075 | A/G | A | 0.326 | -0.05 | 2.3E-04 | -0.08 | 1.8E-09 |
| 16q24 | rs10048146 | [2] | 16 | 85268161 | A/G | G | 0.190 | −0.09 | 1.7E-08 | −0.09 | 1.7E-07 |
| 17q21 | rs1107748 | [3] | 17 | 39129340 | C/T | T | 0.643 | -0.05 | 0.0012 | -0.07 | 1.0E-07 |
|  | rs7220711 | [3] | 17 | 39145491 | A/G | A | 0.663 | -0.05 | 2.1E-04 | -0.08 | 2.2E-08 |
|  | rs1513670 | [3] | 17 | 39162857 | A/G | A | 0.371 | -0.04 | 0.0023 | -0.08 | 2.1E-08 |
| 17q21 | rs228769 | [3] | 17 | 39548711 | C/G | C | 0.800 | -0.07 | 1.0E-05 | -0.08 | 5.8E-08 |
| 17q21 | rs9303521 | [2] | 17 | 41160977 | G/T | T | 0.460 | −0.07 | 5.0E-08 | −0.06 | 8.3E-06 |
| 18q21 | rs884205 | [2] | 18 | 58205837 | G/T | T | 0.270 | −0.08 | 3.8E-08 | −0.04 | 0.0050 |
|  | rs3018362 | [1] | 18 | 58233073 | A/G | A | 0.355 | -0.06 | 8.0E-06 | -0.08 | 5.4E-08 |

1. Styrkarsdottir U, Halldorsson BV, Gretarsdottir S, Gudbjartsson DF, Walters GB, et al. (2008) Multiple genetic loci for bone mineral density and fractures. N Engl J Med 358: 2355-2365.

2. Rivadeneira F, Styrkarsdottir U, Estrada K, Halldorsson BV, Hsu YH, et al. (2009) Twenty bone-mineral-density loci identified by large-scale meta-analysis of genome-wide association studies. Nat Genet 41: 1199-1206.

3. Styrkarsdottir U, Halldorsson BV, Gretarsdottir S, Gudbjartsson DF, Walters GB, et al. (2009) New sequence variants associated with bone mineral density. Nat Genet 41: 15-17.
