# Supplementary material for: European Bone Mineral Density Loci Are Also Associated with BMD in East-Asian Populations
Source: PLoS One. 2010 Oct 7;5(10):e13217. doi: 10.1371/journal.pone.0013217 (PMC2951352; doi:10.1371/journal.pone.0013217)
Supplement: Table S2 — Results for spine BMD for all the SNPs tested in the East-Asian samples. The effect on spine BMD in the East-Asian populations of all SNPs tested in this study. The frequency of the allele that associated with lowered BMD in Europeans is shown along with its effect on spine BMD. A FDR of 0.05, corresponding to a P value threshold of 0.021, was used to determine significance of association. (0.16 MB DOC) [file pone.0013217.s002.doc]

**Table S2. Results for spine BMD for all the SNPs tested in the East-Asian samples**

The effect on spine BMD in the East-Asian populations of all SNPs tested in this study. The frequency of the allele that associated with lowered BMD in Europeans is shown along with its effect on spine BMD. A FDR of 0.05, corresponding to a *P* value threshold of 0.021, was used to determine significance of association.

|  |  |  | **Korea (n = 1,396)** | | | **HongKong-I (n = 3,736)** | | | **Hong Kong-II (n = 785)** | | | **(n = 5,719)** | |  |  |
| --- | --- | --- | --- | --- | --- | --- | --- | --- | --- | --- | --- | --- | --- | --- | --- |
| **Locus** | **SNP** | **allele** | **Effect** | **P value** | **Freq.** | **Effect** | **P value** | **Freq.** | **Effect** | **P value** | **Freq.** | **Effect Asia** | **P value Asia** | **P_het_** | ***I*^2^** |
| 1p36 | rs7524102 | A | -0.09 | 0.041 | 0.824 | -0.08 | 0.0034 | 0.788 | -0.07 | 0.015 | 0.770 | -0.08 | 5.5e-05 | 0.96 | 0.0 |
|  | rs6696981 | G | -0.08 | 0.065 | 0.843 | -0.08 | 0.0024 | 0.793 | -0.12 | 0.0011 | 0.782 | -0.09 | 7.0e-06 | 0.68 | 0.0 |
|  | rs6426749 | G | -0.08 | 0.045 | 0.823 | -0.09 | 0.0007 | 0.792 | -0.08 | 0.008 | 0.780 | -0.09 | 7.5e-06 | 0.96 | 0.0 |
|  | rs7543680 | G | -0.07 | 0.075 | 0.772 | -0.06 | 0.0085 | 0.762 | -0.07 | 0.028 | 0.751 | -0.07 | 0.00036 | 0.99 | 0.0 |
| 1p31 | rs2566755 | A | -0.14 | 0.0010 | 0.796 | -0.06 | 0.021 | 0.767 | -0.06 | 0.045 | 0.760 | -0.07 | 0.00012 | 0.23 | 32.8 |
| 2p21 | rs11898505 | G | -0.04 | 0.26 | 0.848 | 0.00 | 0.52 | 0.905 | 0.02 | 0.63 | 0.910 | -0.00 | 0.54 | 0.76 | 0.0 |
| 3p22 | rs10490823 | G | -0.07 | 0.055 | 0.729 | -0.05 | 0.035 | 0.746 | -0.12 | 0.00095 | 0.750 | -0.07 | 0.00015 | 0.32 | 12.0 |
|  | rs87938 | A | -0.03 | 0.23 | 0.617 | -0.01 | 0.29 | 0.643 | -0.06 | 0.016 | 0.660 | -0.03 | 0.027 | 0.43 | 0.0 |
| 4q22 | rs1471403 | C | -0.05 | 0.11 | 0.707 | -0.05 | 0.016 | 0.652 | -0.04 | 0.085 | 0.650 | -0.05 | 0.0026 | 0.93 | 0.0 |
| 5q14 | rs1366594 | C | -0.02 | 0.27 | 0.610 | -0.01 | 0.37 | 0.576 | -0.02 | 0.24 | 0.580 | -0.01 | 0.18 | 0.92 | 0.0 |
| 6p21 | rs3130340 | T | -0.03 | 0.24 | 0.810 | -0.02 | 0.23 | 0.744 | 0.01 | 0.58 | 0.750 | -0.01 | 0.23 | 0.77 | 0.0 |
| 6q25 | rs9479055 | C | 0.09 | 0.98 | 0.728 | -0.02 | 0.27 | 0.822 | 0.03 | 0.72 | 0.824 | 0.02 | 0.80 | 0.13 | 51.6 |
|  | rs9478223 | C | 0.11 | 0.91 | 0.055 | -0.01 | 0.42 | 0.049 | -0.03 | 0.33 | 0.068 | 0.01 | 0.59 | 0.37 | 0.0 |
|  | rs4870044 | T | 0.04 | 0.79 | 0.778 | -0.03 | 0.17 | 0.830 | -0.04 | 0.20 | 0.832 | -0.02 | 0.23 | 0.41 | 0.0 |
|  | rs1038304 | G | 0.01 | 0.64 | 0.432 | 0.05 | 0.98 | 0.521 | 0.02 | 0.73 | 0.535 | 0.03 | 0.98 | 0.68 | 0.0 |
|  | rs6929137 | A | 0.03 | 0.79 | 0.282 | 0.06 | 1.0 | 0.345 | -0.00 | 0.51 | 0.346 | 0.04 | 0.99 | 0.31 | 14.7 |
|  | rs7751941 | A | -0.51 | 0.0075 | 0.008 | -0.17 | 0.055 | 0.012 | -0.22 | 0.050 | 0.015 | -0.23 | 0.0014 | 0.35 | 4.9 |
|  | rs6900157 | C | 0.06 | 0.91 | 0.313 | 0.08 | 1.0 | 0.372 | 0.00 | 0.51 | 0.389 | 0.05 | 1.0 | 0.14 | 48.3 |
|  | rs2941740 | T | -0.07 | 0.10 | 0.890 | -0.07 | 0.036 | 0.883 | -0.01 | 0.45 | 0.880 | -0.05 | 0.032 | 0.50 | 0.0 |
|  | rs1999805 | C | -0.02 | 0.30 | 0.742 | -0.05 | 0.035 | 0.760 | -0.03 | 0.23 | 0.748 | -0.04 | 0.027 | 0.83 | 0.0 |
|  | rs2504063 | A | -0.03 | 0.29 | 0.806 | -0.02 | 0.35 | 0.808 | -0.04 | 0.15 | 0.800 | -0.03 | 0.12 | 0.93 | 0.0 |
| 7p14 | rs1524058 | T | -0.04 | 0.14 | 0.410 | -0.06 | 0.0028 | 0.439 | -0.05 | 0.029 | 0.440 | -0.06 | 0.00025 | 0.87 | 0.0 |
| 7q21 | rs4729260 | G | -0.03 | 0.33 | 0.132 | -0.01 | 0.35 | 0.141 | -0.16 | 3.0e-05 | 0.130 | -0.07 | 0.0027 | 0.013 | 77.0 |
|  | rs7781370 | T | -0.06 | 0.16 | 0.119 | -0.04 | 0.12 | 0.136 | -0.16 | 3.4e-05 | 0.140 | -0.08 | 0.00017 | 0.066 | 63.2 |
| 8q24 | rs4355801 | A | -0.02 | 0.35 | 0.715 | -0.09 | 0.0008 | 0.779 | -0.08 | 0.030 | 0.795 | -0.07 | 0.00037 | 0.33 | 9.4 |
|  | rs2062377 | T | -0.02 | 0.30 | 0.721 | -0.08 | 0.0022 | 0.222 | -0.06 | 0.033 | 0.220 | -0.06 | 0.00065 | 0.50 | 0.0 |
|  | rs6469792 | C | -0.00 | 0.52 | 0.576 | -0.08 | 0.00055 | 0.632 | -0.10 | 0.0019 | 0.637 | -0.07 | 3.4e-05 | 0.21 | 35.5 |
|  | rs6469804 | A | -0.01 | 0.40 | 0.771 | -0.08 | 0.0029 | 0.813 | -0.10 | 0.008 | 0.823 | -0.07 | 0.00048 | 0.28 | 20.9 |
|  | rs6993813 | C | -0.00 | 0.52 | 0.620 | -0.07 | 0.0043 | 0.673 | -0.09 | 0.0037 | 0.674 | -0.06 | 0.00032 | 0.30 | 17.1 |
| 11p15 | rs7117858 | A | -0.01 | 0.45 | 0.770 | -0.01 | 0.30 | 0.797 | -0.09 | 0.0042 | 0.800 | -0.04 | 0.025 | 0.18 | 41.8 |
| 11p13 | rs16921914 | G | 0.05 | 0.88 | 0.592 | -0.02 | 0.16 | 0.591 | 0.02 | 0.75 | 0.610 | 0.00 | 0.58 | 0.25 | 27.9 |
| 11p11 | rs7932354 | C | -0.01 | 0.41 | 0.317 | -0.03 | 0.13 | 0.305 | -0.00 | 0.54 | 0.310 | -0.02 | 0.18 | 0.79 | 0.0 |
| 11q13 | rs599083 | G | -0.11 | 0.0029 | 0.317 | -0.04 | 0.07 | 0.246 | -0.04 | 0.10 | 0.260 | -0.06 | 0.0014 | 0.30 | 16.8 |
| 12q13 | rs2016266 | A | 0.04 | 0.77 | 0.794 | -0.08 | 0.0055 | 0.837 | 0.02 | 0.68 | 0.840 | -0.03 | 0.12 | 0.051 | 66.3 |
| 13q14 | rs7992970 | A | -0.03 | 0.23 | 0.667 | -0.07 | 0.0029 | 0.682 | -0.10 | 0.0026 | 0.689 | -0.07 | 7.5e-05 | 0.45 | 0.0 |
|  | rs9533090 | T | -0.13 | 0.029 | 0.080 | -0.01 | 0.41 | 0.076 | -0.07 | 0.065 | 0.090 | -0.06 | 0.028 | 0.32 | 11.4 |
|  | rs9594738 | T | -0.14 | 0.0095 | 0.101 | -0.07 | 0.055 | 0.080 | -0.09 | 0.060 | 0.089 | -0.09 | 0.0010 | 0.59 | 0.0 |
|  | rs9533093 | T | -0.04 | 0.17 | 0.418 | 0.00 | 0.53 | 0.413 | 0.01 | 0.56 | 0.414 | -0.01 | 0.39 | 0.65 | 0.0 |
|  | rs10507508 | A | -0.03 | 0.34 | 0.896 | -0.09 | 0.0023 | 0.871 | -0.13 | 0.0036 | 0.864 | -0.09 | 0.00011 | 0.42 | 0.0 |
|  | rs9594751 | T | -0.14 | 0.021 | 0.089 | -0.12 | 0.0085 | 0.055 | -0.10 | 0.070 | 0.069 | -0.12 | 0.00030 | 0.92 | 0.0 |
|  | rs9594759 | T | -0.07 | 0.070 | 0.214 | 0.01 | 0.70 | 0.238 | 0.07 | 0.97 | 0.250 | 0.01 | 0.77 | 0.066 | 63.1 |
| 14q32 | rs2010281 | A | -0.07 | 0.11 | 0.117 | -0.03 | 0.14 | 0.148 | -0.03 | 0.24 | 0.147 | -0.04 | 0.049 | 0.84 | 0.0 |
| 16q24 | rs10048146 | G | 0.00 | 0.53 | 0.323 | -0.05 | 0.035 | 0.274 | -0.03 | 0.20 | 0.290 | -0.03 | 0.04 | 0.62 | 0.0 |
| 17q21 | rs1107748 | T | -0.03 | 0.21 | 0.635 | -0.02 | 0.20 | 0.655 | -0.05 | 0.10 | 0.671 | -0.03 | 0.055 | 0.84 | 0.0 |
|  | rs7220711 | A | -0.03 | 0.22 | 0.650 | -0.01 | 0.34 | 0.696 | -0.05 | 0.09 | 0.711 | -0.03 | 0.09 | 0.67 | 0.0 |
|  | rs1513670 | A | -0.00 | 0.51 | 0.540 | -0.00 | 0.57 | 0.612 | -0.03 | 0.17 | 0.612 | -0.01 | 0.25 | 0.80 | 0.0 |
| 17q21 | rs228769 | C | 0.05 | 0.91 | 0.397 | -0.04 | 0.035 | 0.312 | -0.01 | 0.34 | 0.310 | -0.02 | 0.18 | 0.11 | 54.5 |
| 17q21 | rs9303521 | T | -0.03 | 0.23 | 0.436 | 0.01 | 0.68 | 0.340 | -0.04 | 0.075 | 0.350 | -0.01 | 0.20 | 0.34 | 6.6 |
| 18q21 | rs884205 | T | -0.08 | 0.036 | 0.227 | -0.05 | 0.035 | 0.204 | 0.02 | 0.72 | 0.205 | -0.04 | 0.033 | 0.18 | 42.4 |
|  | rs3018362 | A | -0.00 | 0.53 | 0.708 | -0.01 | 0.32 | 0.778 | -0.05 | 0.10 | 0.754 | -0.02 | 0.15 | 0.66 | 0.0 |
