# Supplementary material for: European Bone Mineral Density Loci Are Also Associated with BMD in East-Asian Populations
Source: PLoS One. 2010 Oct 7;5(10):e13217. doi: 10.1371/journal.pone.0013217 (PMC2951352; doi:10.1371/journal.pone.0013217)
Supplement: Table S3 — Results for hip BMD for all the SNPs tested in the East-Asian samples. The effect on hip BMD in the East-Asian populations of all SNPs tested in this study. The frequency of the allele that associated with lowered BMD in Europeans is shown along with its effect on hip BMD. A FDR of 0.05, corresponding to a P value threshold of 0.025, was used to determine significance of association. (0.16 MB DOC) [file pone.0013217.s003.doc]

**Table S3. Results for hip BMD for all the SNPs tested in the East-Asian samples**

The effect on hip BMD in the East-Asian populations of all SNPs tested in this study. The frequency of the allele that associated with lowered BMD in Europeans is shown along with its effect on hip BMD. A FDR of 0.05, corresponding to a *P* value threshold of 0.025, was used to determine significance of association.

|  |  |  | **Korea (n = 1,396)** | | | **HongKong-I (n = 3,807)** | | | **Hong Kong-II (n = 785)** | | | **(n = 5,988)** | |  |  |
| --- | --- | --- | --- | --- | --- | --- | --- | --- | --- | --- | --- | --- | --- | --- | --- |
| **Locus** | **SNP** | **Allele** | **Effect** | **P value** | **Freq.** | **Effect** | **P value** | **Freq.** | **Effect** | **P value** | **Freq.** | **Effect Asia** | **P value Asia** | **P_het_** | ***I*^2^** |
| 1p36 | rs7524102 | A | -0.11 | 0.012 | 0.824 | -0.11 | 8.0e-05 | 0.789 | -0.09 | 0.0014 | 0.788 | -0.10 | 5.5e-08 | 0.94 | 0.0 |
|  | rs6696981 | G | -0.13 | 0.007 | 0.843 | -0.10 | 0.00016 | 0.793 | -0.15 | 9.0e-05 | 0.793 | -0.12 | 8.0e-09 | 0.59 | 0.0 |
|  | rs6426749 | G | -0.12 | 0.006 | 0.823 | -0.12 | 1.5e-05 | 0.793 | -0.10 | 0.00090 | 0.792 | -0.11 | 4.3e-09 | 0.86 | 0.0 |
|  | rs7543680 | G | -0.08 | 0.034 | 0.772 | -0.08 | 0.0023 | 0.763 | -0.09 | 0.012 | 0.762 | -0.08 | 2.5e-05 | 0.97 | 0.0 |
| 1p31 | rs2566755 | A | -0.15 | 0.00075 | 0.796 | -0.05 | 0.029 | 0.768 | -0.06 | 0.041 | 0.767 | -0.07 | 0.00014 | 0.17 | 42.7 |
| 2p21 | rs11898505 | G | 0.03 | 0.69 | 0.848 | 0.02 | 0.69 | 0.905 | 0.01 | 0.57 | 0.905 | 0.02 | 0.76 | 0.97 | 0.0 |
| 3p22 | rs10490823 | G | -0.07 | 0.055 | 0.729 | -0.06 | 0.0090 | 0.745 | -0.09 | 0.0085 | 0.746 | -0.07 | 0.00013 | 0.80 | 0.0 |
|  | rs87938 | A | -0.06 | 0.07 | 0.617 | -0.04 | 0.065 | 0.643 | -0.03 | 0.14 | 0.643 | -0.04 | 0.0095 | 0.85 | 0.0 |
| 4q22 | rs1471403 | C | 0.00 | 0.5 | 0.707 | -0.06 | 0.0060 | 0.652 | -0.03 | 0.18 | 0.652 | -0.05 | 0.0065 | 0.63 | 0.0 |
| 5q14 | rs1366594 | C | -0.08 | 0.022 | 0.610 | -0.05 | 0.028 | 0.575 | -0.09 | 0.00032 | 0.576 | -0.07 | 1.3e-05 | 0.41 | 0.0 |
| 6p21 | rs3130340 | T | -0.04 | 0.17 | 0.810 | -0.04 | 0.080 | 0.743 | -0.00 | 0.52 | 0.744 | -0.03 | 0.070 | 0.72 | 0.0 |
| 6q25 | rs9479055 | C | 0.10 | 0.99 | 0.728 | -0.05 | 0.037 | 0.822 | 0.04 | 0.81 | 0.822 | 0.01 | 0.61 | 0.0093 | 78.6 |
|  | rs9478223 | C | -0.11 | 0.1 | 0.055 | -0.16 | 0.0031 | 0.049 | -0.00 | 0.52 | 0.049 | -0.09 | 0.0070 | 0.21 | 36.1 |
|  | rs4870044 | T | 0.09 | 0.98 | 0.778 | -0.07 | 0.0075 | 0.830 | 0.01 | 0.54 | 0.830 | -0.02 | 0.24 | 0.0073 | 79.7 |
|  | rs1038304 | G | -0.01 | 0.42 | 0.432 | 0.03 | 0.88 | 0.522 | 0.02 | 0.73 | 0.521 | 0.02 | 0.86 | 0.75 | 0.0 |
|  | rs6929137 | A | 0.02 | 0.72 | 0.282 | 0.04 | 0.97 | 0.345 | -0.02 | 0.32 | 0.345 | 0.02 | 0.92 | 0.38 | 0.0 |
|  | rs7751941 | A | -0.29 | 0.08 | 0.008 | -0.06 | 0.28 | 0.012 | -0.37 | 0.0043 | 0.012 | -0.19 | 0.0075 | 0.19 | 39.7 |
|  | rs6900157 | C | 0.04 | 0.85 | 0.313 | 0.05 | 0.98 | 0.372 | -0.02 | 0.32 | 0.372 | 0.03 | 0.96 | 0.25 | 28.8 |
|  | rs2941740 | T | -0.11 | 0.029 | 0.890 | -0.07 | 0.034 | 0.883 | -0.07 | 0.046 | 0.883 | -0.08 | 0.0011 | 0.81 | 0.0 |
|  | rs1999805 | C | -0.01 | 0.42 | 0.742 | -0.08 | 0.0014 | 0.758 | -0.02 | 0.27 | 0.760 | -0.05 | 0.0047 | 0.28 | 22.1 |
|  | rs2504063 | A | -0.05 | 0.15 | 0.806 | -0.08 | 0.026 | 0.808 | -0.04 | 0.14 | 0.808 | -0.05 | 0.011 | 0.73 | 0.0 |
| 7p14 | rs1524058 | T | -0.04 | 0.14 | 0.410 | -0.07 | 0.0015 | 0.440 | -0.02 | 0.26 | 0.439 | -0.05 | 0.0020 | 0.37 | 0.0 |
| 7q21 | rs4729260 | G | -0.02 | 0.37 | 0.132 | -0.03 | 0.16 | 0.141 | -0.17 | 9.0e-06 | 0.141 | -0.08 | 0.00038 | 0.017 | 75.6 |
|  | rs7781370 | T | -0.03 | 0.32 | 0.119 | -0.04 | 0.12 | 0.136 | -0.16 | 1.9e-05 | 0.136 | -0.08 | 0.00025 | 0.039 | 69.1 |
| 8q24 | rs4355801 | A | 0.06 | 0.92 | 0.715 | -0.05 | 0.049 | 0.779 | -0.06 | 0.065 | 0.779 | -0.03 | 0.10 | 0.069 | 62.6 |
|  | rs2062377 | T | 0.05 | 0.87 | 0.721 | -0.04 | 0.080 | 0.779 | -0.04 | 0.085 | 0.222 | -0.02 | 0.11 | 0.16 | 44.7 |
|  | rs6469792 | C | 0.05 | 0.89 | 0.576 | -0.06 | 0.0085 | 0.633 | -0.13 | 8.5e-05 | 0.632 | -0.05 | 0.00095 | 0.0029 | 82.9 |
|  | rs6469804 | A | 0.04 | 0.79 | 0.771 | -0.03 | 0.18 | 0.814 | -0.09 | 0.018 | 0.813 | -0.03 | 0.10 | 0.12 | 52.1 |
|  | rs6993813 | C | 0.03 | 0.8 | 0.620 | -0.04 | 0.050 | 0.673 | -0.12 | 0.00030 | 0.673 | -0.05 | 0.0055 | 0.013 | 77.1 |
| 11p15 | rs7117858 | A | -0.08 | 0.028 | 0.770 | -0.04 | 0.10 | 0.796 | -0.10 | 0.0018 | 0.797 | -0.07 | 0.00033 | 0.34 | 6.8 |
| 11p13 | rs16921914 | G | 0.04 | 0.86 | 0.592 | -0.00 | 0.53 | 0.591 | -0.01 | 0.34 | 0.591 | 0.00 | 0.56 | 0.50 | 0.0 |
| 11p11 | rs7932354 | C | -0.01 | 0.45 | 0.317 | -0.04 | 0.060 | 0.305 | 0.03 | 0.84 | 0.305 | -0.01 | 0.30 | 0.21 | 36.1 |
| 11q13 | rs599083 | G | -0.05 | 0.12 | 0.317 | -0.03 | 0.12 | 0.246 | -0.07 | 0.0085 | 0.246 | -0.05 | 0.0033 | 0.59 | 0.0 |
| 12q13 | rs2016266 | A | 0.06 | 0.88 | 0.794 | -0.05 | 0.050 | 0.837 | 0.01 | 0.61 | 0.837 | -0.01 | 0.33 | 0.14 | 49.4 |
| 13q14 | rs7992970 | A | -0.06 | 0.07 | 0.667 | -0.04 | 0.050 | 0.681 | -0.06 | 0.055 | 0.682 | -0.05 | 0.0037 | 0.86 | 0.0 |
|  | rs9533090 | T | -0.04 | 0.28 | 0.080 | 0.00 | 0.54 | 0.075 | -0.05 | 0.17 | 0.076 | -0.02 | 0.21 | 0.73 | 0.0 |
|  | rs9594738 | T | -0.01 | 0.41 | 0.101 | -0.05 | 0.12 | 0.079 | -0.07 | 0.13 | 0.080 | -0.05 | 0.065 | 0.80 | 0.0 |
|  | rs9533093 | T | 0.05 | 0.9 | 0.418 | 0.01 | 0.65 | 0.413 | 0.02 | 0.72 | 0.413 | 0.02 | 0.88 | 0.67 | 0.0 |
|  | rs10507508 | A | -0.15 | 0.008 | 0.896 | -0.04 | 0.09 | 0.872 | -0.11 | 0.011 | 0.871 | -0.08 | 0.00075 | 0.25 | 28.9 |
|  | rs9594751 | T | -0.01 | 0.41 | 0.089 | -0.05 | 0.18 | 0.054 | -0.02 | 0.38 | 0.055 | -0.03 | 0.18 | 0.92 | 0.0 |
|  | rs9594759 | T | 0.03 | 0.73 | 0.214 | -0.00 | 0.55 | 0.236 | 0.07 | 0.97 | 0.238 | 0.02 | 0.86 | 0.28 | 20.7 |
| 14q32 | rs2010281 | A | -0.04 | 0.23 | 0.117 | -0.03 | 0.15 | 0.147 | -0.02 | 0.36 | 0.148 | -0.03 | 0.10 | 0.94 | 0.0 |
| 16q24 | rs10048146 | G | -0.09 | 0.011 | 0.323 | -0.07 | 0.0055 | 0.274 | -0.02 | 0.27 | 0.274 | -0.05 | 0.001 | 0.26 | 26.6 |
| 17q21 | rs1107748 | T | -0.07 | 0.04 | 0.635 | -0.02 | 0.17 | 0.656 | -0.01 | 0.40 | 0.655 | -0.03 | 0.05 | 0.50 | 0.0 |
|  | rs7220711 | A | -0.08 | 0.025 | 0.650 | -0.03 | 0.14 | 0.696 | -0.01 | 0.36 | 0.696 | -0.03 | 0.032 | 0.45 | 0.0 |
|  | rs1513670 | A | -0.01 | 0.35 | 0.540 | -0.05 | 0.021 | 0.613 | -0.03 | 0.16 | 0.612 | -0.04 | 0.015 | 0.75 | 0.0 |
| 17q21 | rs228769 | C | 0.04 | 0.82 | 0.397 | -0.06 | 0.005 | 0.312 | -0.00 | 0.54 | 0.312 | -0.02 | 0.075 | 0.063 | 63.8 |
| 17q21 | rs9303521 | T | -0.00 | 0.52 | 0.436 | -0.03 | 0.10 | 0.339 | -0.00 | 0.53 | 0.340 | -0.02 | 0.17 | 0.73 | 0.0 |
| 18q21 | rs884205 | T | -0.09 | 0.025 | 0.227 | -0.03 | 0.18 | 0.204 | 0.06 | 0.94 | 0.205 | -0.01 | 0.24 | 0.033 | 70.6 |
| 18q21 | rs3018362 | A | 0.02 | 0.66 | 0.708 | 0.03 | 0.82 | 0.776 | -0.04 | 0.17 | 0.778 | 0.01 | 0.67 | 0.42 | 0.0 |
