# Supplementary material for: European Bone Mineral Density Loci Are Also Associated with BMD in East-Asian Populations
Source: PLoS One. 2010 Oct 7;5(10):e13217. doi: 10.1371/journal.pone.0013217 (PMC2951352; doi:10.1371/journal.pone.0013217)
Supplement: Table S4 — Difference in effect on BMD between Europeans and East-Asians. The effect on hip BMD and spine BMD in populations of European descent and of East-Asian descent of SNPs tested in this study. The frequency of the allele that associated with lowered BMD in Europeans is shown for Europeans and for East-Asians along with its effect on spine BMD and hip BMD in both ethnicities. The P value comparing the effect between the two ethnicities is shown. Effects deemed different using a FDR of 0.05 corresponds to a P value threshold of 0.012 for Spine BMD and 0.008 for Hip BMD. (0.13 MB DOC) [file pone.0013217.s004.doc]

**Table S4. Difference in effect on BMD between Europeans and East-Asians**

The effect on hip BMD and spine BMD in populations of European descent and of East-Asian descent of SNPs tested in this study. The frequency of the allele that associated with lowered BMD in Europeans is shown for Europeans and for East-Asians along with its effect on spine BMD and hip BMD in both ethnicities. The *P* value comparing the effect between the two ethnicities is shown. Effects deemed different using a FDR of 0.05 corresponds to a P value threshold of 0.012 for Spine BMD and 0.008 for Hip BMD.

|  |  |  |  |  | **Spine BMD** | | | | | **Hip BMD** | | | | |
| --- | --- | --- | --- | --- | --- | --- | --- | --- | --- | --- | --- | --- | --- | --- |
| **Locus** | **SNP** | **Allele** | **Freq Europe** | **Freq Asia** | **Effect Europe** | **P value Europe** | **Effect Asia** | **P value Asia** | ***P*** | **Effect Europe** | **P value Europe** | **Effect Asia** | **P value Asia** | ***P*** |
| 1p36 | rs7524102 | A | 0.830 | 0.794 | -0.11 | 7.0E-09 | -0.08 | 5.5e-05 | 0.21 | -0.15 | 1.2E-16 | -0.10 | 5.5e-08 | 0.075 |
|  | rs6696981 | G | 0.864 | 0.803 | -0.12 | 2.4E-08 | -0.09 | 7.0e-06 | 0.35 | -0.14 | 2.5E-12 | -0.12 | 8.0e-09 | 0.50 |
|  | rs6426749 | G | 0.830 | 0.798 | -0.11 | 3.8E-09 | -0.09 | 7.5e-06 | 0.36 | -0.08 | 1.5E-07 | -0.11 | 4.3e-09 | 0.20 |
|  | rs7543680 | G | 0.772 | 0.763 | -0.07 | 9.3E-06 | -0.07 | 0.00036 | 0.91 | -0.10 | 1.0E-10 | -0.08 | 2.5e-05 | 0.45 |
| 1p31 | rs2566755 | A | 0.790 | 0.773 | -0.10 | 3.1E-12 | -0.07 | 0.00012 | 0.22 | -0.10 | 1.1E-11 | -0.07 | 0.00014 | 0.19 |
| 2p21 | rs11898505 | G | 0.660 | 0.892 | -0.07 | 6.3E-08 | -0.00 | 0.54 | 0.023 | -0.03 | 0.030 | 0.02 | 0.76 | 0.10 |
| 3p22 | rs10490823 | G | 0.460 | 0.742 | -0.05 | 2.8E-04 | -0.07 | 0.00015 | 0.39 | -0.06 | 9.4E-06 | -0.07 | 0.00013 | 0.63 |
|  | rs87938 | A | 0.450 | 0.639 | -0.04 | 3.1E-04 | -0.03 | 0.027 | 0.72 | -0.07 | 3.4E-09 | -0.04 | 0.0095 | 0.13 |
| 4q22 | rs1471403 | C | 0.660 | 0.665 | -0.07 | 5.7E-08 | -0.05 | 0.0026 | 0.30 | -0.06 | 2.0E-06 | -0.05 | 0.0065 | 0.53 |
| 5q14 | rs1366594 | C | 0.450 | 0.584 | 0.01 | 0.66 | -0.01 | 0.18 | 0.38 | -0.09 | 1.1E-12 | -0.07 | 1.3e-05 | 0.28 |
| 6p21 | rs3130340 | T | 0.795 | 0.760 | -0.10 | 1.2E-07 | -0.01 | 0.23 | 0.0017 | -0.05 | 0.0065 | -0.03 | 0.070 | 0.44 |
| 6q25 | rs9479055 | C | 0.355 | 0.800 | -0.08 | 6.2E-07 | 0.02 | 0.80 | 0.00024 | -0.08 | 3.1E-08 | 0.01 | 0.61 | 0.00087 |
|  | rs9478223 | C | 0.104 | 0.053 | -0.14 | 2.5E-10 | 0.01 | 0.59 | 0.00069 | -0.11 | 1.1E-06 | -0.09 | 0.0070 | 0.73 |
|  | rs4870044 | T | 0.286 | 0.818 | -0.11 | 6.6E-13 | -0.02 | 0.23 | 0.00039 | -0.09 | 8.0E-10 | -0.02 | 0.24 | 0.0040 |
|  | rs1038304 | G | 0.472 | 0.502 | -0.09 | 1.9E-10 | 0.03 | 0.98 | 2.5e-08 | -0.08 | 4.0E-10 | 0.02 | 0.86 | 4.4e-06 |
|  | rs6929137 | A | 0.299 | 0.330 | -0.10 | 1.6E-10 | 0.04 | 0.99 | 3.2e-09 | -0.08 | 1.5E-08 | 0.02 | 0.92 | 5.2e-06 |
|  | rs7751941 | A | 0.217 | 0.011 | -0.09 | 5.7E-08 | -0.23 | 0.0014 | 0.073 | -0.09 | 1.3E-07 | -0.19 | 0.0075 | 0.21 |
|  | rs6900157 | C | 0.316 | 0.360 | -0.09 | 4.2E-09 | 0.05 | 1 | 1.4e-09 | -0.09 | 2.2E-09 | 0.03 | 0.96 | 2.3e-07 |
|  | rs2941740 | T | 0.570 | 0.884 | -0.07 | 9.3E-09 | -0.05 | 0.032 | 0.43 | -0.07 | 9.1E-10 | -0.08 | 0.0011 | 0.82 |
|  | rs1999805 | C | 0.440 | 0.754 | -0.09 | 2.2E-08 | -0.04 | 0.027 | 0.040 | -0.06 | 1.2E-04 | -0.05 | 0.0047 | 0.73 |
|  | rs2504063 | A | 0.400 | 0.806 | -0.08 | 1.5E-08 | -0.03 | 0.12 | 0.053 | -0.06 | 4.4E-06 | -0.05 | 0.011 | 0.79 |
| 7p14 | rs1524058 | T | 0.400 | 0.432 | -0.07 | 5.2E-09 | -0.06 | 0.00025 | 0.50 | -0.04 | 0.0014 | -0.05 | 0.0020 | 0.76 |
| 7q21 | rs4729260 | G | 0.320 | 0.137 | -0.08 | 9.5E-10 | -0.07 | 0.0027 | 0.58 | -0.09 | 5.4E-11 | -0.08 | 0.00038 | 0.64 |
|  | rs7781370 | T | 0.340 | 0.132 | -0.07 | 5.5E-09 | -0.08 | 0.00017 | 0.58 | -0.08 | 2.9E-11 | -0.08 | 0.00025 | 0.97 |
| 8q24 | rs4355801 | A | 0.536 | 0.766 | -0.09 | 5.2E-10 | -0.07 | 0.00037 | 0.39 | -0.07 | 5.3E-07 | -0.03 | 0.10 | 0.074 |
|  | rs2062377 | T | 0.560 | 0.339 | -0.09 | 5.7E-15 | -0.06 | 0.00065 | 0.20 | -0.06 | 1.7E-07 | -0.02 | 0.11 | 0.099 |
|  | rs6469792 | C | 0.529 | 0.619 | -0.11 | 1.1E-15 | -0.07 | 3.4e-05 | 0.087 | -0.10 | 3.1E-13 | -0.05 | 0.00095 | 0.042 |
|  | rs6469804 | A | 0.518 | 0.805 | -0.12 | 7.4E-15 | -0.07 | 0.00048 | 0.067 | -0.08 | 2.5E-09 | -0.03 | 0.10 | 0.040 |
|  | rs6993813 | C | 0.504 | 0.660 | -0.12 | 1.8E-14 | -0.06 | 0.00032 | 0.020 | -0.09 | 3.3E-11 | -0.05 | 0.0055 | 0.050 |
| 11p15 | rs7117858 | A | 0.800 | 0.791 | 0.04 | 0.0050 | -0.04 | 0.025 | 0.0012 | -0.09 | 2.7E-09 | -0.07 | 0.00033 | 0.32 |
| 11p13 | rs16921914 | G | 0.730 | 0.594 | -0.08 | 1.0E-08 | 0.00 | 0.58 | 0.00010 | -0.04 | 0.0050 | 0.00 | 0.56 | 0.051 |
| 11p11 | rs7932354 | C | 0.710 | 0.309 | -0.06 | 2.4E-05 | -0.02 | 0.18 | 0.051 | -0.07 | 1.5E-08 | -0.01 | 0.30 | 0.0045 |
| 11q13 | rs599083 | G | 0.310 | 0.264 | -0.07 | 1.7E-07 | -0.06 | 0.0014 | 0.52 | -0.05 | 2.0E-04 | -0.05 | 0.0033 | 0.98 |
| 12q13 | rs2016266 | A | 0.680 | 0.827 | -0.07 | 5.2E-08 | -0.03 | 0.12 | 0.078 | -0.05 | 3.0E-04 | -0.01 | 0.33 | 0.11 |
| 13q14 | rs7992970 | A | 0.777 | 0.679 | -0.11 | 2.7E-10 | -0.07 | 7.5e-05 | 0.11 | -0.08 | 8.2E-06 | -0.05 | 0.0037 | 0.23 |
|  | rs9533090 | T | 0.500 | 0.078 | -0.12 | 4.6E-23 | -0.06 | 0.028 | 0.056 | -0.04 | 6.0E-04 | -0.02 | 0.21 | 0.63 |
|  | rs9594738 | T | 0.568 | 0.086 | -0.17 | 2.0E-21 | -0.09 | 0.0010 | 0.025 | -0.10 | 1.9E-08 | -0.05 | 0.065 | 0.13 |
|  | rs9533093 | T | 0.808 | 0.414 | -0.11 | 5.4E-11 | -0.01 | 0.39 | 1.5e-05 | -0.04 | 0.038 | 0.02 | 0.88 | 0.021 |
|  | rs10507508 | A | 0.947 | 0.876 | -0.17 | 3.6E-07 | -0.09 | 0.00011 | 0.064 | -0.13 | 5.3E-05 | -0.08 | 0.00075 | 0.22 |
|  | rs9594751 | T | 0.265 | 0.065 | -0.08 | 3.7E-07 | -0.12 | 0.00030 | 0.32 | -0.07 | 2.1E-05 | -0.03 | 0.18 | 0.31 |
|  | rs9594759 | T | 0.622 | 0.234 | -0.13 | 1.1E-16 | 0.01 | 0.77 | 1.1e-08 | -0.07 | 2.1E-06 | 0.02 | 0.86 | 0.00020 |
| 14q32 | rs2010281 | A | 0.326 | 0.140 | -0.05 | 2.3E-04 | -0.04 | 0.049 | 0.73 | -0.08 | 1.8E-09 | -0.03 | 0.10 | 0.081 |
| 16q24 | rs10048146 | G | 0.190 | 0.288 | -0.09 | 6.0E-08 | -0.03 | 0.040 | 0.017 | -0.09 | 4.9E-07 | -0.05 | 0.0010 | 0.15 |
| 17q21 | rs1107748 | T | 0.643 | 0.652 | -0.05 | 0.0012 | -0.03 | 0.055 | 0.37 | -0.07 | 1.0E-07 | -0.03 | 0.05 | 0.068 |
|  | rs7220711 | A | 0.663 | 0.687 | -0.05 | 2.1E-04 | -0.03 | 0.09 | 0.28 | -0.08 | 2.2E-08 | -0.03 | 0.032 | 0.052 |
|  | rs1513670 | A | 0.371 | 0.595 | -0.04 | 0.0023 | -0.01 | 0.25 | 0.25 | -0.08 | 2.1E-08 | -0.04 | 0.015 | 0.064 |
| 17q21 | rs228769 | C | 0.800 | 0.331 | -0.07 | 1.0E-05 | -0.02 | 0.18 | 0.019 | -0.08 | 5.8E-08 | -0.02 | 0.075 | 0.012 |
| 17q21 | rs9303521 | T | 0.460 | 0.364 | -0.07 | 5.0E-08 | -0.01 | 0.20 | 0.0080 | -0.06 | 8.3E-06 | -0.02 | 0.17 | 0.052 |
| 18q21 | rs884205 | T | 0.270 | 0.210 | -0.08 | 3.8E-08 | -0.04 | 0.033 | 0.095 | -0.04 | 0.0050 | -0.01 | 0.24 | 0.30 |
|  | rs3018362 | A | 0.355 | 0.759 | -0.06 | 8.0E-06 | -0.02 | 0.15 | 0.11 | -0.08 | 5.4E-08 | 0.01 | 0.67 | 0.00042 |
